# Fecal Microbiota Transplantation for Weight and Glycemic Control of Obesity as Well as the Associated Metabolic Diseases: Meta-Analysis and Comprehensive Assessment

**DOI:** 10.3390/life13071488

**Published:** 2023-06-30

**Authors:** Diangeng Hu, Jianxin Zhao, Hao Zhang, Gang Wang, Zhennan Gu

**Affiliations:** 1 State Key Laboratory of Food Science and Resources, Jiangnan University, Wuxi 214122, Chinawanggang@jiangnan.edu.cn (G.W.); 2 School of Food Science and Technology, Jiangnan University, Wuxi 214122, China; 3 National Engineering Research Center for Functional Food, Jiangnan University, Wuxi 214122, China; 4 (Yangzhou) Institute of Food Biotechnology, Jiangnan University, Yangzhou 225004, China

**Keywords:** fecal microbiota transplantation (FMT), weight, blood glucose, obesity, obesity-associated metabolic diseases

## Abstract

**Objectives:** An analysis of the weight and blood glucose management associated with fecal microbiota transplantation (FMT) as well as metabolic diseases associated with FMT was conducted by the authors in order to provide clinical recommendations regarding the treatment of nonalcoholic fatty liver disease (NAFLD) and type 2 diabetes mellitus (T2DM). **Methods:** We searched PubMed, Embase, and the Cochrane Library for papers that were published between the creation of the database and October 2022. We reviewed research that investigated how FMT affected weight and glycemic management in cases of obesity and metabolic conditions that are related to obesity. Studies that were published more than once, lacked the entire text, included insufficient information, or were impossible to extract data from were excluded. Additionally, case reports, reviews, and systematic reviews were excluded from the analysis. In order to analyze the data, STATA 15.1 was used. **Outcomes:** When we combined all of our findings, we discovered that pooled outcomes showed that weight levels (WMD equals −4.77, 95%CI: −7.40~−2.14), BMI levels (WMD equals −1.59, 95%CI: −2.21~−0.97), HOMA-IR (WMD equals −0.79, 95%CI: −1.57~−0.00), and HbA1c (WMD equals −0.65, 95%CI: −0.75~−0.55) after FMT treatment were significantly lower than before treatment. However, FMT treatment may have no effect on glucose and insulin levels in obese patients at fasting and related metabolic diseases. Additionally, subgroup analysis outcomes found that FMT significantly reduced fasting blood glucose in people with diabetes. **Conclusions:** As a weight loss and glycemic control therapy, FMT helps to prevent and treat metabolic problems linked to obesity, and is a viable alternative to bariatric surgery for patients who do not wish to undergo the procedure.

## 1. Introduction

Obesity refers to a certain degree of being obviously overweight and where the fat layer is too thick, in terms of a state of body fat, especially where there is too much triglyceride accumulation. It is not simply due to weight gain but also fat tissue that accumulates in the body as a result of obesity. Obesity is on the rise globally, according to the World Health Organization Statistics, which show that by 2017, nearly 2.1 billion adults in the world were overweight, accounting for 43.10% of the total population, and more than 700 million were obese [1]. Obesity is not simply a matter of body size management. More importantly, obesity not only leads to a decreased quality of life but also poses serious health risks [2]. In recent decades, the number of obese individuals has increased significantly [1]. It is currently very difficult to find effective medication therapies for metabolic illnesses, such as type 2 diabetes, heart disease, and nonalcoholic fatty liver disease, which are associated with obesity [2,3]. At present, there are not many weight-loss methods applied in clinical practice, and people have sought a variety of weight-loss methods. For people with extreme obesity, surgery can be used to lose weight in clinic [4]. As a result of bariatric surgery, weight loss can be achieved effectively and metabolic diseases associated with obesity can be significantly reduced [3,5]. While bariatric surgery aids in weight loss and reduces health issues, it is invasive and associated with a high rate of illness and death [4]. In addition, bariatric surgery alters how much food the body eats by altering the secretion of ghrelin, bile acids, gut hormones, and so on. This process also affects the central prefrontal cortex and dopaminergic signaling pathways, which may increase the body’s sensitivity to other rewards, such as alcohol [6]. Therefore, these side effects also limit the widespread use of bariatric surgery in the treatment of obesity. Experts recommend adjuvant drug therapy for obese persons with a body mass index (BMI) of 30 kg/m^2^ or higher with obesity-related diseases [7]. Weight-loss drugs used in obesity are primarily appetite suppressants that target the center and other drugs that target peripheral tissues to a lesser extent, reducing body weight as a result of a change in the balance between intake and the expenditure of energy [8]. However, diet pills can also cause pain, insomnia, anxiety, and gastrointestinal adverse reactions, and are prone to relapse after withdrawal [9,10,11,12]. The discovery of new, effective treatment options for obesity and related metabolic disorders is of vital importance [13,14,15].

At present, the international implementation of FMT is still not standardized [16]. Donors are usually strictly screened before transplantation of FMT, and the selection of child donors is usually made from their mothers, siblings, or healthy children [17]. Then, according to the recipient’s own condition and disease type, the corresponding intestinal preparation is made, and antibiotics or laxatives are used to clean the intestinal tract before transplantation [18,19,20]. The transplanted materials are divided into fresh feces and frozen feces [21]. Transplantation routes can be divided into oral coprofecal capsules, upper GI tract (gastroscopy, nasogastric tube, nasojejunal tube, or percutaneous gastro-jejunostomy tube infusion), and lower GI tract diameter (colonoscopy, colonic tube insertion, or enema) [22]. Colonoscopy is the current first-line approach, which is usually performed in a single FMT and can be repeated in initial non-responders [23]. The first successful use of FMT in children with Clostridioides and separate difficile infection (CDI) was reported in 2010 [24]. Subsequently, there is more experience of using FMT in treating children with CDI. In 2013, the Food and Drug Administration included FMT in its treatment guidelines for recurrent CDI [25]. Other scholars have found that FMT can be used to treat children with metabolic diseases, allergic diseases, autism spectrum disorders, and other extra-intestinal diseases. For metabolic diseases, the transplantation of fecal microbiota is an innovative method for restoring gut microbiota diversity and balance [26,27,28,29,30]. Microbiome dysbiosis has been linked to gut flora control in studies, which contributes to type 2 diabetes, nonalcoholic fatty liver disease, and obesity. In recent years, researchers have explored the potential use of FTM in the treatment of metabolic conditions such as obesity. As of yet, there is no scientific evidence to support the hypothesis that FTM affects glycemic control and weight in individuals suffering from obesity and other metabolic diseases. As part of the study, this research employed a thorough investigation and meta-analysis of how individuals with FTM, NAFLD, T2DM, and other related metabolic disorders manage their weight and blood glucose levels in order to enhance clinical therapy [6,7,8,9,10].

## 2. Approaches

### 2.1. Criteria for Including and Excluding Literature

Inclusion criteria: This study is a single-arm study; it reports on fat mass and glycemic control in metabolic illnesses including obesity. The sole language used for the research is English.

There are a number of exclusion criteria, including repetition of publication; studies without full texts, incomplete information, or difficulty extracting data; animal experiments; case studies; as well as review articles and systematic reviews.

### 2.2. Search Technique

For this meta-assessment, we investigated PubMed, Embase, as well as the Cochrane Library from time the databases were created until October 2022. The search terms are “FMT”, “obesity”, “faecal microbiota transplantation”, “faecal bacteria transplantation”, “faecal transplantation”, and “faecal bacteriotherapy”. This disorder is also known as “obesity”, “type-2 diabetes mellitus”, “type 2 diabetes”, “nonalcoholic liver problems”, or “NAFLD”.

### 2.3. Data Extraction and Literature Screening

The literature was searched, screened, and collected independently by two researchers. If there is a dispute or disagreement, a decision is reached after consulting with a third party or bargaining with them. All of the above information was gathered from the papers, including the author, publication year, country, sample size, age, gender, and outcomes including weight, body mass index, fasting blood sugar (mmol/L), fasting insulin, assessment of insulin resistance using the homeostatic model, and hemoglobin A1C.

### 2.4. Literature Quality Assessment

To evaluate the strength of the evidence in each study, we used the methodological indicator for non-randomized studies (MINORS) scale. Two independent researchers conducted the study. A score between 0 and 2 is assigned to each of the 12 items for a total of 24 points. From the age of 9 to 16, the quality of the study was rated moderate, and from the age of 17 to 24, the study was rated excellent.

### 2.5. Synthesis of Data and Statistical Evaluation

In order to determine whether there is heterogeneity in the data, STATA 15.1 was used (Stata Crop LP, College Station, TX, USA). [14] I2 and Q tests were used to check for heterogeneity. When the heterogeneity test outcomes in *p* ≥ 0.1 and I2 ≤ 50%, then both studies are equivalent, and a fixed effects model is used in the combined analysis. Sensitivity analysis was conducted to determine why the outcomes differed if *p* < 0.1 or I2 > 50 percent. The random effects model is used if the groups still differ significantly, or quit if trying to combine the findings and turn to descriptive analysis. The assessment of the publishing bias employed a funnel plot.

## 3. Outcomes

### 3.1. Outcomes of Literature Study

**Figure 1:**
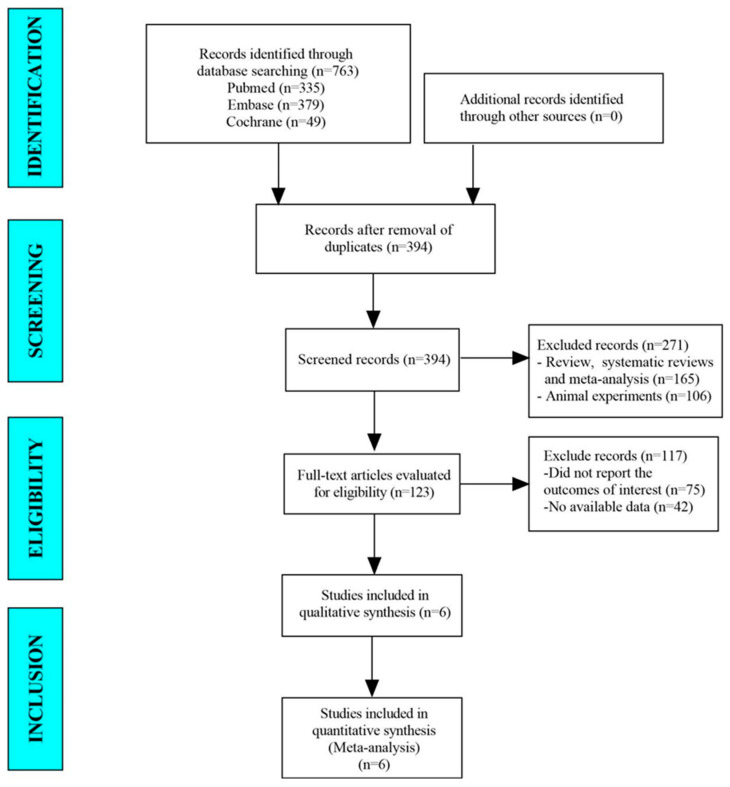
The flow diagram for selecting studies.

PubMed, Embase, and the Cochrane Library’s databases, which included 763 papers, were examined in this meta-analysis. There were 394 studies remaining after the duplicates were removed. In total, 123 studies were found thanks to the titles and abstracts. In the end, the meta-analysis looked at 6 papers (Figure 1).

### 3.2. Baseline Attributes and Evaluation of the Listed Studies’ Quality

This meta-analysis comprised a total of six publications. There were 130 patients in the sample, of which a total of 92 patients were males and 38 patients were females. Patients from the United States participated in three trials, whereas participants in the other three studies were from China. The range of ages was 42.5 to 57.3 on average. Average fasting blood glucose concentrations ranged from 5.19 to 8.5. Since every MINORS score was higher than 16, the included material was of a moderate or high quality (Table 1).

### 3.3. Meta-Analysis Outcomes

#### 3.3.1. Weight

**Figure 2:**
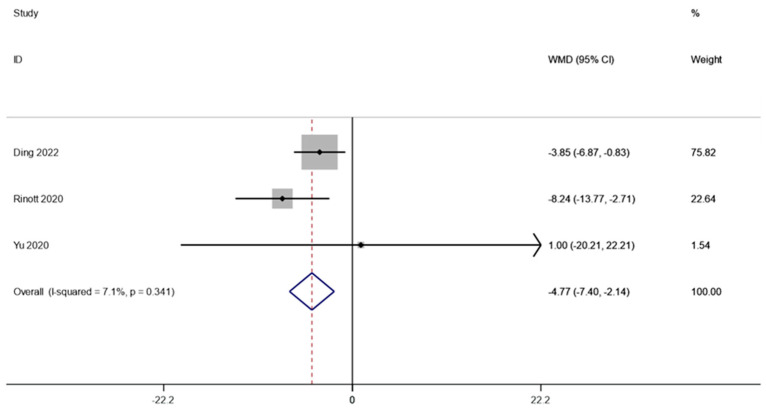
Weight in obesity and associated metabolic diseases following FMT therapy.

Three studies involving 67 patients examined the weight of individuals with obesity and metabolic disorders. A fixed effects model was employed to do a meta-analysis since there was not much of a difference between the groups (I2 is equal to 7.1 percent, *p* is equal to 0.341). The combined data demonstrated that weight levels were significantly lower following FMT therapy compared to the baseline (WMD is equal to −4.77, 95 percent CI: −7.40 ~−2.14, *p* is equal to 0.000; Figure 2), indicating that FMT therapy helps reduce body weight in obese patients and those with metabolic diseases related to obesity.

#### 3.3.2. BMI

**Figure 3:**
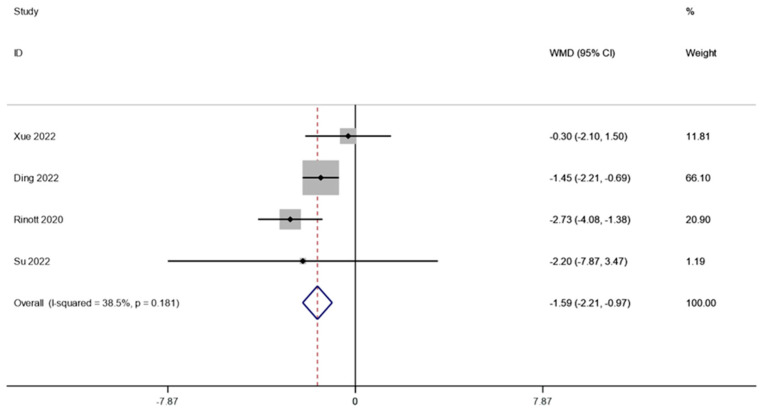
BMI in obesity and associated metabolic diseases following FMT.

The BMI of obese individuals and the associated metabolic illnesses were examined in four investigations (including 105 patients). A fixed effects model was employed to perform a meta-analysis since there was not much of distinction between the cohorts (I2 is equal to 38.5 percent, *p* is equal to 0.181). The combined data demonstrated that BMI levels were significantly lower after FMT therapy than they were before therapy (WMD is equal to −1.59, 95 percent CI: −2.21~−0.97, *p* is equal to 0.000; Figure 3), indicating that FMT therapy helps reduce BMI levels in obese patients and those with metabolic diseases related to obesity.

#### 3.3.3. Fasting Blood Glucose

**Figure 4:**
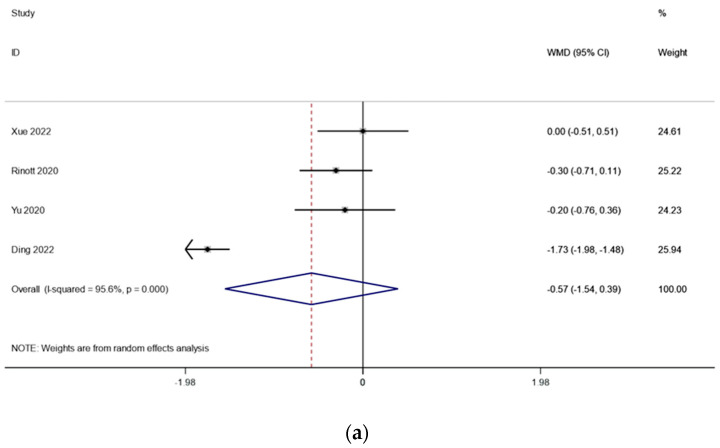

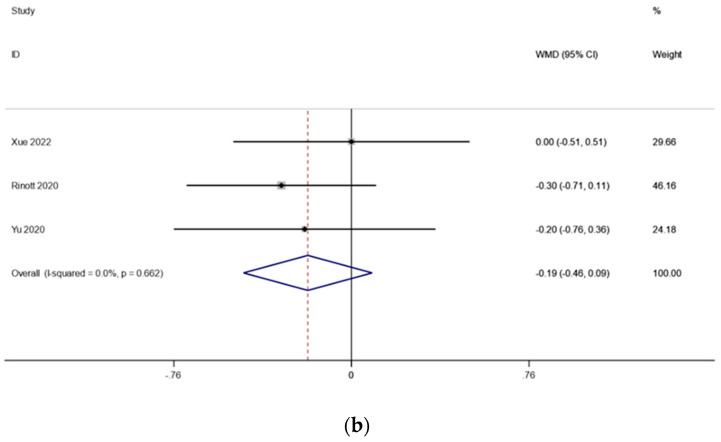
(**a**) Fasting blood glucose in obesity and associated metabolic diseases after FMT (before sensitivity analysis); (**b**) fasting blood glucose in obesity and associated metabolic diseases following FMT (after sensitivity analysis).

There were three studies (including 114 patients) that looked at the fasting blood sugar levels in people with obesity and metabolic diseases related to obesity. The outcomes changed because of what Ding et al. found. Because there was very little heterogeneity (I2 is equal to 95.6 percent, p is equal to 0.000) (Figure 4a), we conducted a sensitivity analysis and found that the study by Ding et al. [17] had an effect on the outcomes. There was significantly reduced heterogeneity (*I2 is equal to 0.0 percent, p is equal to 0.662*) when this literature was excluded, and the meta-analysis was carried out using a fixed effects model. the fasting blood glucose levels before and after FMT treatment, according to the pooled outcomes, did not change substantially from one another (WMD is equal to −0.19, 95 percent CI: −0.46~0.36, *p* is equal to 0.184; Figure 4b), suggesting that FMT treatment may have no effect on fasting blood glucose in obese patients and those with metabolic diseases related to obesity.

#### 3.3.4. Fasting Insulin

**Figure 5:**
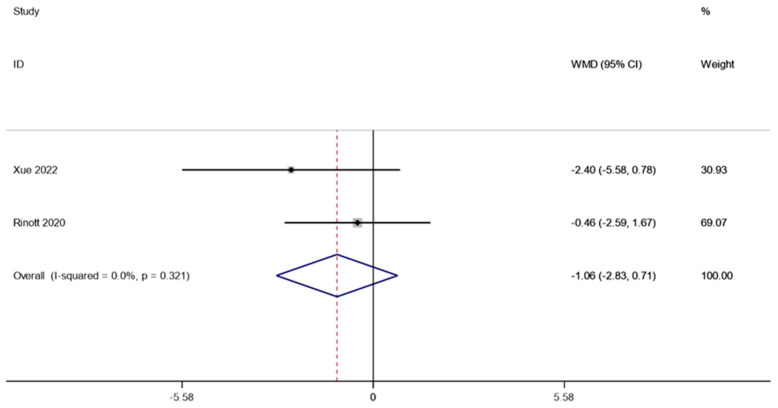
Fasting insulin in obesity and associated metabolic diseases following FMT.

Following FMT, two research studies (involving 91 patients) examined how fasting insulin affected obesity and other metabolic illnesses. A fixed effect model was utilized to perform a meta-analysis since there was no discernible heterogeneity (I2 is equal to 0.0 percent, *p* is equal to 0.321). According to the combined data, the level of fasting insulin after FMT therapy did not vary substantially from the level before therapy (WMD is equal to −1.06, 95 percent CI: −2.83~0.71, *p* is equal to 0.241; Figure 5), suggesting that patients suffering from obesity and related metabolic diseases may not benefit from FMT treatment in terms of fasting insulin levels.

#### 3.3.5. HOMA-IR

**Figure 6:**
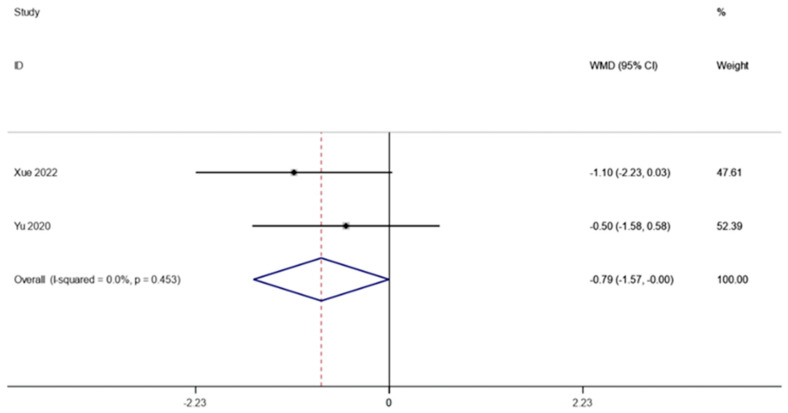
HOMA-IR in obesity and associated metabolic diseases following FMT.

Following FMT, the HOMA-IR in obesity and associated metabolic disorders was observed in two investigations (including 59 patients). A fixed effects model was employed to perform a meta-analysis since there was not much of a difference between the groups (I2 is equal to 0.0 percent, *p* is equal to 0.453). HOMA-IR was significantly lower after FMT therapy than it was before, according to the pooled data (WMD is equal to −0.79, 95 percent CI: −1.57~−0.00, *p* is equal to 0.049; Figure 6), indicating that FMT therapy helps reduce HOMA-IR in obese patients and those with metabolic diseases related to obesity.

#### 3.3.6. HbA1c

**Figure 7:**
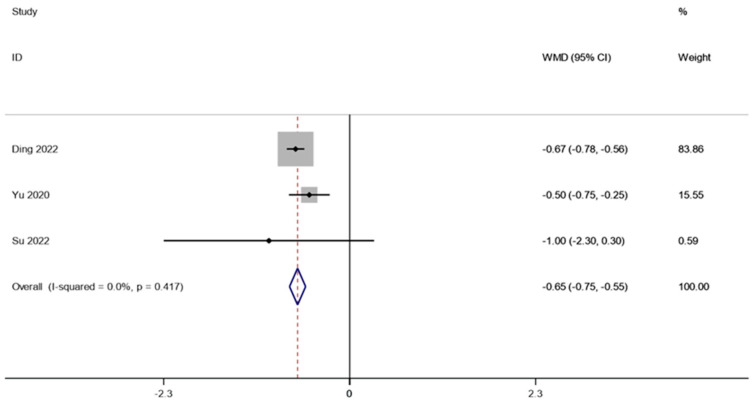
HbA1c in obesity and associated metabolic diseases following FMT.

There were two studies (involving 28 patients) that reported the HbA1c in obesity and related metabolic diseases after FMT. A fixed effects model was employed to conduct a meta-analysis since there was not much of a difference between the groups (I2 is equal to 0.0 percent, *p* is equal to 0.417). A1c was considerably lower after FMT therapy than it was before (WMD is equal to −0.65, 95 percent CI: −0.75~−0.55, *p* is equal to 0.000; Figure 7), indicating that FMT therapy helps reduce HbA1c in obese patients and those with metabolic diseases related to obesity.

### 3.4. Analysis of Subgroups

**Figure 8:**
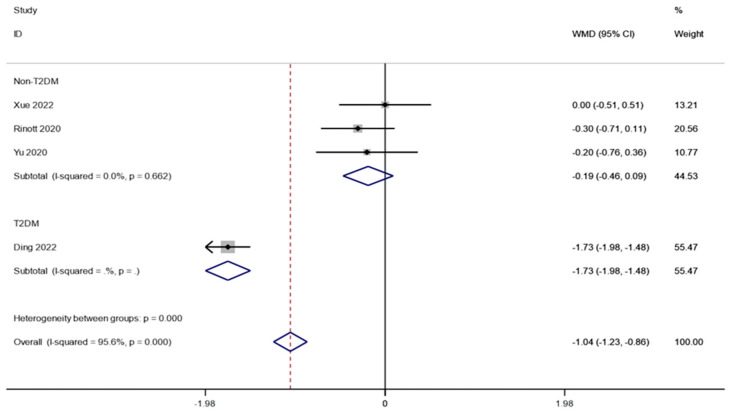
Subgroup analysis for fasting blood glucose in obesity and associated metabolic diseases following FMT.

Even though there were significant differences in the blood glucose measurement, sensitivity analysis revealed that research from Ding et al. had a significant impact on the outcomes. However, looking at baseline characteristics, we found that Ding et al.’s study included diabetic populations, while other studies included samples of normal glycemic populations. Therefore, we further based our subgroup analyses on whether or not people with diabetes were identified. It was found that FMT significantly reduced fasting blood glucose in people with diabetes (WMD is equal to −1.73, 95 percent CI:−1.98~−1.48, *p* is equal to 0.000; Figure 8).

### 3.5. Sensitivity Evaluation

**Figure 9:**
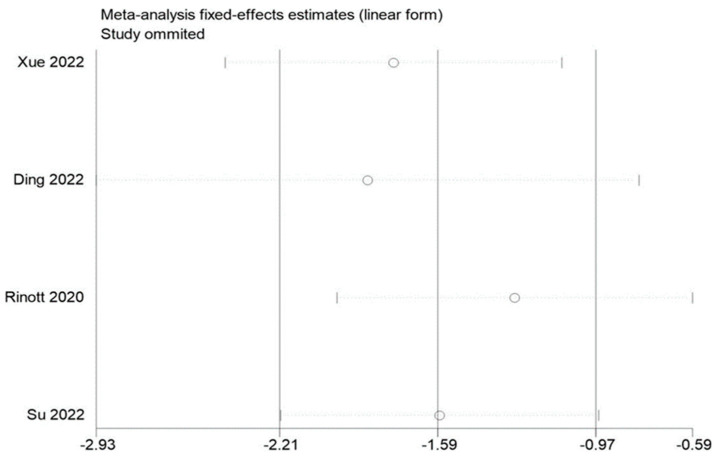
Sensitivity analysis of BMI in obesity and associated metabolic diseases following FMT.

**Figure 10:**
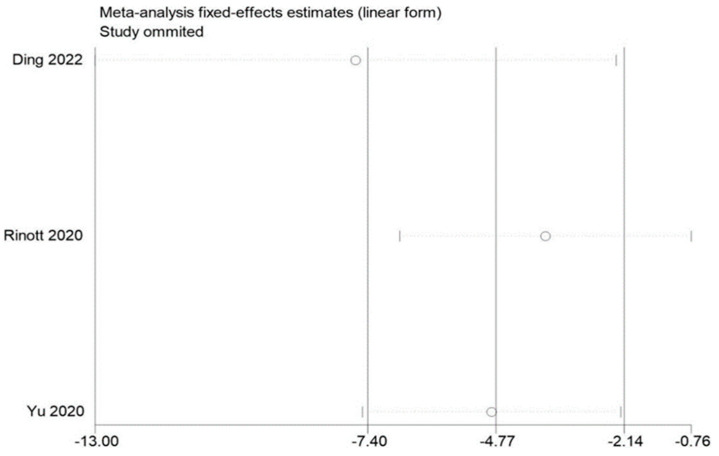
Sensitivity analysis of weight in obesity and associated metabolic diseases following FMT.

**Figure 11:**
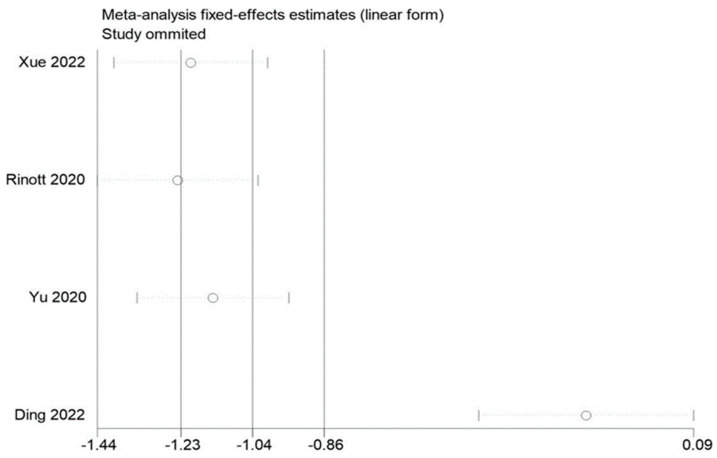
Sensitivity analysis of fasting blood glucose in obesity and associated metabolic diseases following FMT.

By removing each of the included studies one at a time and performing a summary assessment of those that were still available, we performed a sensitivity analysis. Our investigation revealed that, although the other findings were mostly constant, the study by Ding et al. had an effect on the analysis of fasting blood glucose. In addition, no significant influence on the outcomes was found in the indicators of other studies, indicating that there is a good deal of stability and reliability in the outcomes of other research. The findings of the sensitivity analysis are displayed in Figure 9, Figure 10 and Figure 11.

### 3.6. Publication Bias

**Figure 12:**
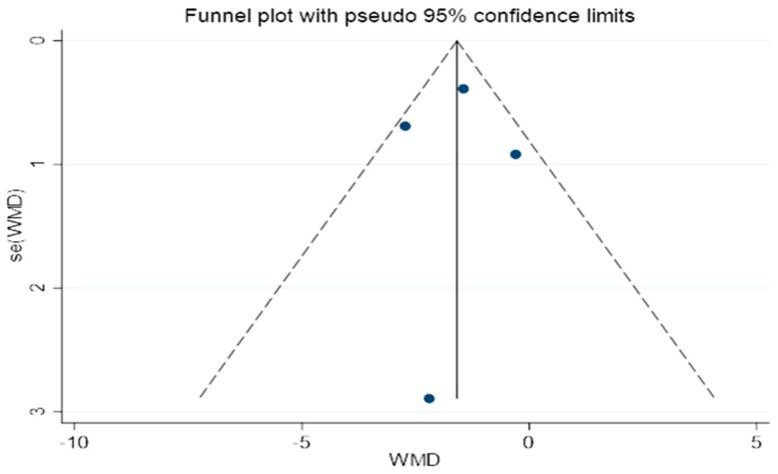
A funnel plot for determining publication bias.

An illustration of the funnel plots can be found in Figure 12. A bias in publication is not evident in this study, as shown by the fact that the two funnel plots all had the same shape.

## 4. Discussion

Over the past 50 years, throughout the world, the amount of obese individuals has continued to rise, making obesity a major public health issue. Obesity is not simply a matter of body size management [31]. More importantly, obesity not only leads to a decreased quality of life but also poses serious health risks. Overweight and obesity are associated with several metabolic diseases (including type 2 diabetes and nonalcoholic fatty liver disease), as well as cardiovascular diseases (such as hypertension, myocardial infarction, and stroke), as well as certain cancers (such as liver, colon, breast, etc.) [32]. As a result, obesity imposes a heavy burden on the health of individuals, the public health system, and the social economy. As overweight and obesity are on the rise, the epidemic level is high, and the whole population is affected by the situation; preventing obesity and controlling it is still faced with great challenges [33]. However, existing treatments for obesity are still limited and come with health risks. Bariatric surgery and weight-loss drugs help people get rid of the trouble of obesity by reducing appetite, reducing the body’s absorption of lipids, and other means, at the same time; they also bury dangerous hidden dangers in people’s body, such as increased blood pressure, rapid heart rate, anorexia, insomnia, abnormal liver function, depression, diarrhea, and other harmful side effects [34]. Although changing one’s lifestyle in terms of diet and rhythm is considered to be the safest and most effective way to prevent the occurrence of obesity, the effect is limited and difficult to maintain [35]. Obesity and obesity-related metabolic diseases require the development of new, effective treatment strategies. FMT, which involves replenishing the gut microbiota with healthy human feces, is a successful way to treat diseases brought on by intestinal dysbiosis. It is presently the established standard of care for the therapy of recurrent Clostridioides difficile infection [36]. The gut microbiota and metabolic processes have been linked in many research papers, including FMT from thin persons being found to lower obesity and undesirable metabolic characteristics [37]. We evaluated participants’ body weight, BMI, fasting blood glucose, fasting insulin, and HOMA-IR, as well as HbA1C in six studies that looked at the effects of FMT on weight and glycemic management in obesity and other metabolic disorders.

The first findings of this research indicated that weight and BMI were much lower following FMT therapy than they had been. Glucagon-like peptide 1 (GLP1) may be released by the gut microbiota via a route involving SCFA and receptors that are G protein-coupled, which makes you feel full, aids in weight loss, and promotes the emptying of the stomach [38,39]. Additionally, the gut microbiota alters the routes used by bile acids, which impacts how lipids are broken down [40]. This implies that FMT aids in weight loss in obese individuals and the treatment of obesity may be possible with this method.

In addition, the study found that blood glucose levels were not statistically significantly affected by FMT. This may be due to the fact that the people included in the study were all individuals with normal blood glucose. Therefore, we further conducted subgroup analyses of people with diabetes. It was revealed that FMT greatly reduced fasting blood glucose in people with diabetes. However, because Ding et al. only conducted one research [41,42,43,44], which explored the impact of FMT on blood glucose among people suffering from T2DM, the objectivity of the outcomes was challenged. The combined data also revealed that the level of fasting insulin before and after FMT treatment did not change substantially from one another. This may possibly be because FMT only affects insulin levels in diabetic patients; more clinical studies are required to examine how FMT affects insulin levels in both healthy individuals and diabetic patients. Notably, the pooled outcomes showed that HOMA-IR and HbA1c after FMT therapy were significantly lower than before therapy. This outcome is consistent with that of a clinical experiment conducted by Vrieze et al. [9] in which the participants either obtained FMT from their own waste or that of a thin donor (BMI 23 kg/m^2^). The insulin sensitivity of individuals who finished the lean FMT rose. The outcomes suggest that FMT may help control the level of glucose in people with obesity and the metabolic diseases that come with it. FMT was most commonly associated with nausea, and serious adverse events were rare [45,46,47,48]. A small number of children had fever, allergy, and other adverse events, but they were mild and transient [49,50,51]. Of concern, on 13 June 2019, the FDA issued an important warning that FMT may present a serious or life-threatening risk of infection, which may be associated with multidrug-resistant bacteria. Medical workers can avoid risks by standardizing FMT treatment and strictly managing procedures. FMT is a challenging new technique, and its safety needs to be repeatedly demonstrated in future F, M, and T therapies [52].

There are several limitations to this study. Firstly, concerns regarding the impartiality of the combined findings are raised by the limited number of research studies that were included in this analysis, and for the validity of our findings to continue to be validated; new available literature will need to be considered in the future. Secondly, there may be differences between healthy and diabetic people in the analysis of blood glucose and insulin, which may lead to heterogeneity. In the future, there is a need for clinical trials to determine whether FMT has a differential effect on blood glucose levels between healthy individuals and diabetics.

## 5. Conclusions

As a weight loss and glycemic control therapy, FMT helps to prevent and treat metabolic problems linked to obesity, and it is a viable alternative to bariatric surgery for patients who do not wish to undergo the procedure.

## Figures and Tables

**Table 1:** Qualitative assessment of the included studies based on their baseline characteristics.

Author	Year	Study Design	Country	Sample Size	Sex (Male/Female)	Age	BMI	Weight (kg)	Fasting Blood Glucose (mmol/L)	MINORS Score
Jessica [15]	2020	Single-arm	USA	11	10/1	44.5 ± 14.4	41.1 ± 5.0	/	5.19 ± 0.8	16
Xue [16]	2022	Single-arm	China	47	25/22	57.3 ± 13.4	27.7 ± 4.5	/	5.3 ± 1.2	16
Ding [17]	2022	Single-arm	China	11	4/7	55.7 ± 3.8	25.0 ± 0.9	70.3 ± 3.6	8.5 ± 0.4	17
Rinott [18]	2020	Single-arm	USA	44	42/2	53.1 ± 10.0	30.9 ± 3.5	93.7 ± 14.1	5.7 ± 1.1	18
Yu [19]	2020	Single-arm	USA	12	8/4	42.5 ± 8.4	38.8 ± 6.7	110.0 ± 26.0	4.8 ± 0.4	17
Su [20]	2022	Single-arm	China	5	3/2	57.0 ± 13.2	25.2 ± 5.0	/	7.1 ± 1.3	17

## Data Availability

Datasets will be made available upon reasonable request by the corresponding author.
